# Mycobiota associated with diseased Schrenk’s spruce in mountain forest ecosystems of Kyrgyzstan

**DOI:** 10.3389/ffunb.2026.1970334

**Published:** 2026-10-28

**Authors:** Tinatin Doolotkeldieva, Batma Zhusupova

**Affiliations:** Plant Protection Research Centre, Kyrgyz National Agrarian University Named After K.I.Skraybin, Bishkek, Kyrgyzstan

**Keywords:** biosphere reserve, decline of spruce trees, forest health, fungal community, infected plant tissues, morphological and molecular characterisation of fungi

## Abstract

Schrenk’s spruce (*Picea schrenkiana*) is a dominant coniferous tree species in the Western Tian Shan forests and plays an important ecological role in maintaining mountain forest ecosystems in Kyrgyzstan. In recent years, the Sary-Chelek Biosphere Reserve has seen a significant increase in diseased and declining spruce trees, raising concerns about forest health and ecosystem stability. The present study aimed to investigate the fungal communities associated with diseased roots, stems, and needles of Schrenk’s spruce in the Sary-Chelek Biosphere Reserve, Kyrgyzstan. We identified fungal isolates from symptomatic plant tissues using morphological characteristics, ITS (5.8S rRNA) sequencing, and species-specific molecular markers. Mycological analysis revealed a diverse fungal community associated with diseased spruce tissues. *Penicillium* and *Talaromyces* species were predominantly isolated from infected roots. *Penicillium* species were most commonly associated with needle lesions (approximately 50%), followed by *Alternaria* (20%), *Curvularia* (20%), and *Didymella* species (10%). *Fusarium* species were dominant in the bark tissues of diseased stems. The results demonstrate that multiple fungal taxa are involved in the decline of Schrenk’s spruce in the Sary-Chelek Biosphere Reserve. This study provides the first integrated morphological and molecular characterization of fungi associated with declining *P. schrenkiana* in this region and contributes to a better understanding of forest disease dynamics in the mountain ecosystems of Central Asia.

## 1 Introduction

Established in 1959, the Sary-Chelek Nature Reserve (41°52′N 71°59′E) is located in the Aksy District of Jalal-Abad Region, Kyrgyz Republic (**Figure 1**). Spanning 23, 868 hectares along the eastern slope of the Chatkal Ridge, the reserve encompasses a range of landscapes—from foothill steppes to snow-capped peaks and swift rivers.

**Figure 1:**
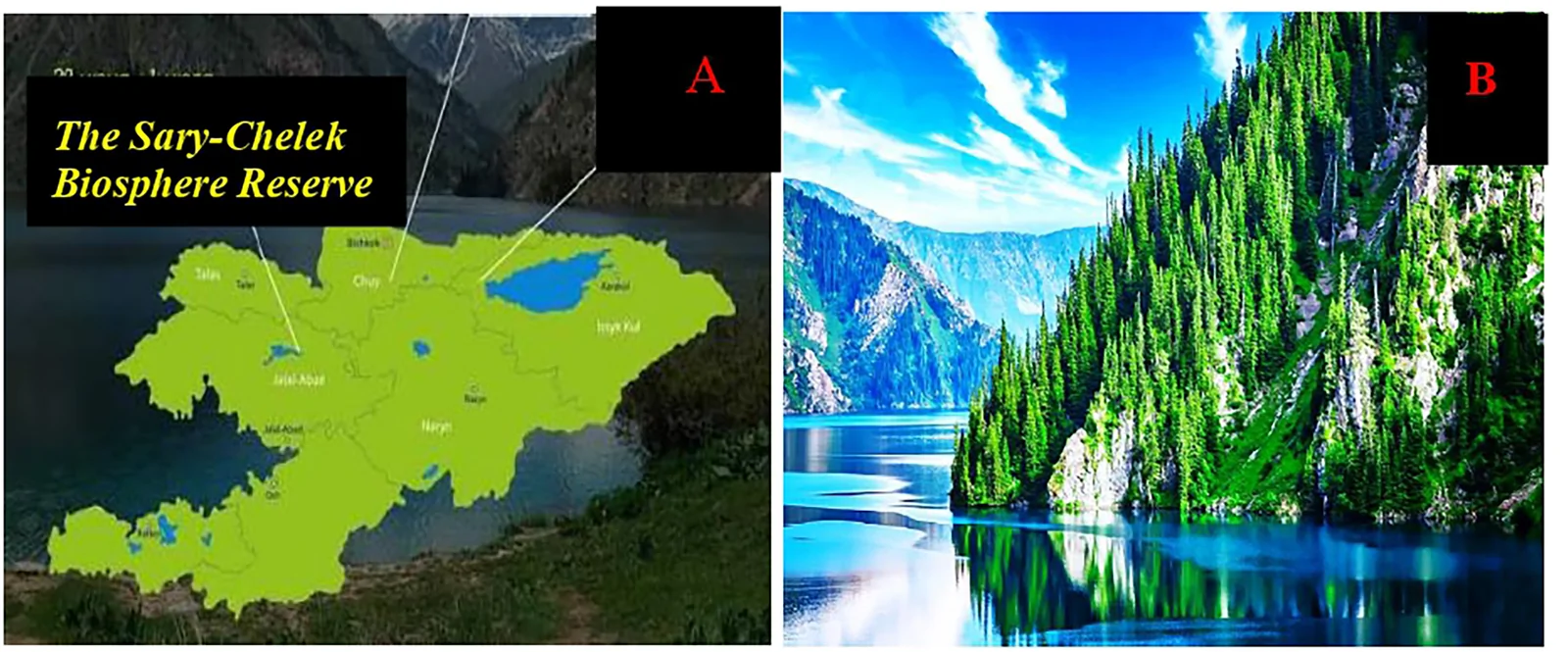
**(A)** Map of Kyrgyzstan highlighting the location of the Sary-Chelek Biosphere Reserve. **(B)** Scenic view of conifer forests lining the shoreline of Sary-Chelek Lake. (https://dwc.kg/places/ozero-sary-chelek-sary-chelekskij-zapovednik/#images-2).

### 1.1 Current threats to coniferous forest species.

Over the past three to five years, a local forester has observed significant drought stress across extensive stands of century-old trees—a trend that continues to spread into new areas each year. Conifers are experiencing complete needle loss and a sustained decline in overall health. Notably, the bark retains moisture up to 0.5 metres in height, while above two metres it peels away, exposing desiccated wood (**Figure 2**). The number of diseased pine trees in Sary-Chelek’s forests has increased sharply, making these one of the four primary factors affecting ecosystem dynamics, alongside pests, fire, and adverse environmental conditions.

**Figure 2:**
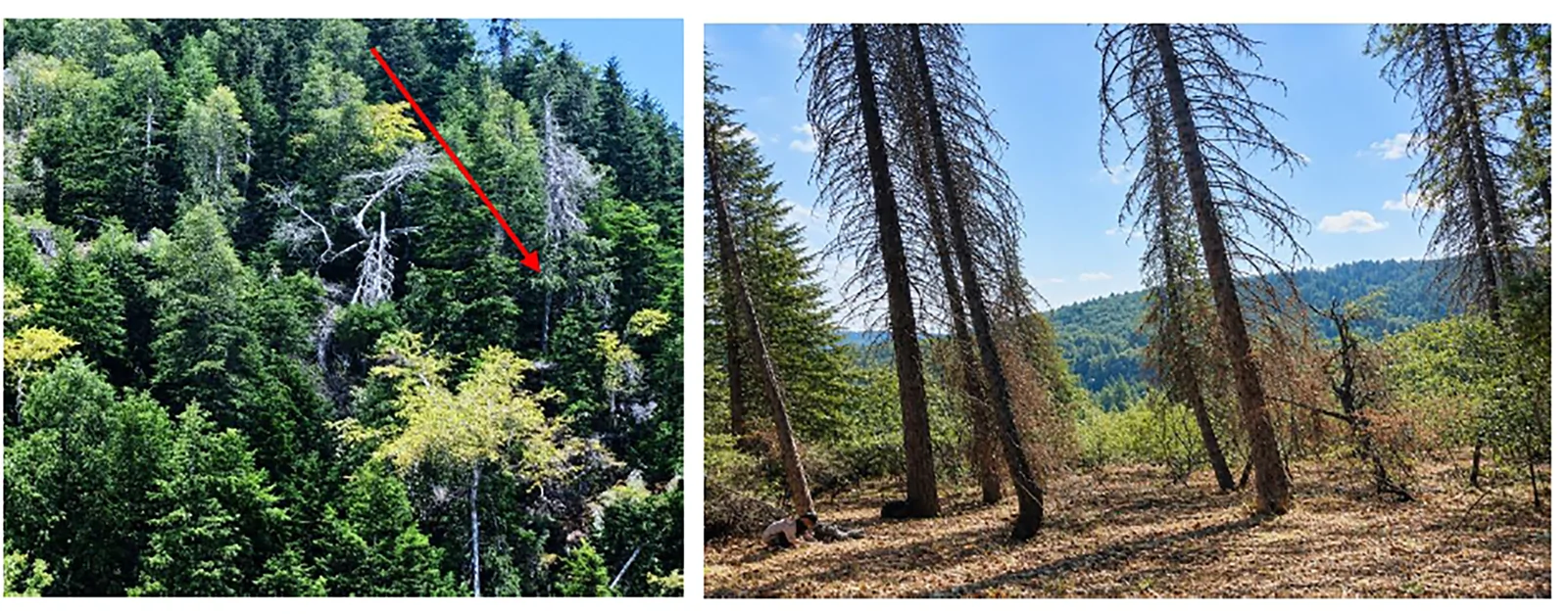
Dried Schrenk's spruce (*Picea schrenkiana*) trees in a protected area.

### 1.2 Drivers of conifer forest decline and the role of fungal communities

Coniferous forests provide a wide range of essential ecosystem services, including long-term carbon sequestration, hydrological regulation, soil stabilisation, climate regulation, and biodiversity conservation. Beyond their ecological importance, coniferous forests support regional economies through timber production, non-timber forest resources, and ecosystem-based tourism. Their long lifespan and structural complexity create habitats for diverse assemblages of plants, fungi, animals, and microorganisms, making them indispensable components of global forest ecosystems (Boa, 2003).

In recent decades, however, coniferous forests have experienced an unprecedented increase in tree decline and mortality across many regions. Widespread forest dieback has been documented throughout Europe, North America, and Asia, where prolonged drought, increasing temperatures, altered precipitation regimes, insect outbreaks, and emerging diseases have substantially reduced forest resilience. These stressors rarely act independently; rather, they interact synergistically, weakening tree defence systems and increasing susceptibility to colonization by opportunistic and pathogenic microorganisms. As a consequence, forest decline has become one of the most pressing challenges for sustainable forest management and biodiversity conservation under ongoing climate change (Talley et al., 2002; Lee et al., 2014; Gomez-Gallego et al., 2022; Desprez-Loustau et al., 2016).

Among the biological agents involved in forest decline, fungi occupy a central role. Numerous fungal species can cause root rot, stem cankers, vascular dysfunction, wood decay, and needle diseases, while many others act as secondary invaders that accelerate tissue degradation in physiologically weakened trees (Shen et al., 2022). Increasing evidence indicates that tree mortality generally results from complex interactions among environmental stress, insect infestation, and diverse fungal communities rather than from infection by a single pathogenic species (Tomao et al., 2020). Consequently, understanding the composition and ecological functions of fungal communities associated with diseased trees has become a major research priority in contemporary forest pathology (Crous et al., 2017; Siddique and Unterseher, 2016; Forkuo and Borz, 2026).

Forest trees host complex fungal communities composed of pathogenic, endophytic, saprotrophic, and mycorrhizal species that collectively influence host health, nutrient cycling, wood decomposition, and ecosystem functioning. Numerous factors determine the composition of these communities, including host species and genotype, tree age, physiological status, climatic conditions, geographic location, soil properties, and interactions with surrounding vegetation. In advanced stages of infection, the combined effects of fungal invasion and physiological stress frequently result in irreversible decline and eventual tree mortality. Disease development rarely involves a single fungal species; instead, multiple taxa often coexist within symptomatic tissues, forming disease complexes that evolve as host decline progresses (Talley et al., 2002; Lee et al., 2014; Gomdola et al., 2022).

Under stable environmental conditions, these fungal assemblages contribute to ecosystem functioning and forest resilience. However, environmental disturbances may disrupt community structure, favour opportunistic pathogens, and alter the balance between beneficial and pathogenic microorganisms (Crous et al., 2017; Siddique and Unterseher, 2016; Gutierrez-Flores et al., 2020).

Pathogenic fungi are responsible for a wide spectrum of diseases affecting coniferous trees, including needle blight, shoot dieback, stem cankers, vascular discoloration, root rot, and wood decay. Disease symptoms often begin with chlorosis and needle necrosis, followed by progressive crown thinning, branch dieback, bark deterioration, and extensive colonization of woody tissues (Braekke and Sahin, 2002; Bednářová et al., 2013). Forest decline is further intensified by interactions among fungi, wood-boring insects, and other associated organisms. Bark beetles and longhorn beetles frequently act as vectors of fungal propagules, facilitating pathogen dispersal and introducing fungi into weakened host tissues in the emergence and spread of pine rot disease (Steiner and Buhrer, 1934; Mamiya and Kiyohara, 1972; Nickle et al., 1981; Maehara et al., 2005; Hyun et al., 2007; Zhao et al., 2014; Hirata et al., 2017). In several coniferous forest ecosystems, disease complexes involving fungal pathogens, bark beetles, and wood-inhabiting nematodes have caused extensive tree mortality, particularly in East Asia (Donald et al., 2016; Zhao, 2008; Cang-sang, 2011). These examples show that the ecological consequences of forest decline arise from interactions among multiple organisms rather than from individual pathogens alone. Advances in fungal systematics and molecular biology have substantially improved our understanding of fungal diversity associated with declining forest trees. Traditional isolation techniques remain valuable for recovering viable fungal cultures and assessing their biological characteristics; however, cultivation-based methods often underestimate total fungal diversity because slow-growing or unculturable taxa may remain undetected (Roberts, 1998). The incorporation of DNA-based identification, particularly sequencing of the internal transcribed spacer (ITS) region together with protein-coding markers such as translation elongation factor 1-alpha (TEF1-α), has greatly enhanced taxonomic resolution and enabled more accurate characterization of fungal communities associated with diseased trees (Xu et al., 2016; Meyer et al., 2019).

Despite considerable progress in understanding fungal communities associated with declining coniferous forests in Europe, North America, and East Asia, comparable information from the mountain forests of Central Asia remains extremely limited. In particular, little is known about the diversity, taxonomic composition, and ecological roles of fungi colonizing diseased *P. schrenkiana* in the Western Tien Shan. This lack of baseline information limits our understanding of forest decline processes in one of the most biologically important mountain regions of Central Asia and constrains the development of effective conservation and forest health management strategies.

### 1.3 Fungal communities associated with pine wilt disease

A diverse assemblage of fungi is consistently associated with pine wilt disease (PWD), contributing to disease development, nematode proliferation, and vector transmission. Among the most frequently reported taxa are saprophytic members of the phylum Ascomycota, particularly *Penicillium*, *Aspergillus*, and *Trichoderma*, which have been isolated from virtually all tissues of wilted pine trees in China, Japan, Korea, Portugal, and the United States (Kobayashi et al., 1974, 1975; Wingfield, 1985, 1987). In addition to these ubiquitous saprophytes, blue-stain and ophiostomatoid fungi, including *Ceratocystis*, *Ophiostoma*, and *Leptographium* (formerly *Verticicladiella*), are commonly recovered from infected trees and are regarded as key components of the pine wilt disease complex.These fungi maintain close ecological relationships with insect vectors, particularly *Monochamus* spp. Longhorn beetles transport fungal propagules while colonizing weakened or dying trees, introducing them into host tissues through feeding wounds and oviposition galleries (Wingfield, 1985, 1987). This interaction is mutually beneficial: fungal colonization facilitates beetle development by improving nutritional conditions for larvae, whereas the beetles promote fungal dispersal over long distances. Simultaneously, *Bursaphelenchus xylophilus* enters a mycophagous phase, feeding extensively on fungal hyphae, which supports rapid nematode reproduction and accelerates disease progression. Consequently, pine wilt disease is increasingly recognized as a multipartite interaction involving the nematode, insect vector, fungal associates, and susceptible host trees.

Among the fungal associates, blue-stain fungi such as *Ophiostoma minus* play a particularly important role in the epidemiology of PWD. Experimental studies in Japan demonstrated that colonization of artificial pupal chambers by *O. minus* significantly enhances the transmission efficiency of *B. xylophilus* by *Monochamus alternatus* (Maehara and Futai, 2000). Moreover, the composition of the fungal community strongly influences nematode population dynamics. Rapid multiplication of *B. xylophilus* has been observed in the presence of *O. minus*, *Macrophoma* spp., and *Trichoderma* spp., whereas colonization by *Verticillium* spp. or certain *Trichoderma* species may suppress nematode populations (Stirling and Smith, 1998; Maehara and Futai, 2000; Maehara, 2008). These findings indicate that fungal species differ substantially in their capacity to promote or inhibit nematode development.

Similar interactions have been documented in China, where *Sporothrix* species have been shown to enhance both the survival of *B. xylophilus* and the fitness of its vector, *M. alternatus* (Zhao et al., 2013). Under favorable environmental conditions, external symptoms of pine wilt disease typically become visible approximately three weeks after nematode invasion, while infected trees frequently die within one to three months.

Endophytic and opportunistic fungi also form an important component of the pine microbiome. Studies of healthy and declining *Pinus thunbergii* identified species of *Pestalotiopsis*, *Nigrospora*, *Cladosporium*, and *Phomopsis* in both healthy and diseased trees, with relatively stable community composition throughout the year (Kuroda and Ito, 1992). Although these fungi are not considered primary causal agents of pine wilt disease, they may influence host physiology, compete with pathogenic fungi, or participate in wood decomposition during later stages of disease development.

Investigations of declining pine trees have further demonstrated that fungal communities extend well beyond blue-stain fungi. In addition to *Ophiostoma* species, wilted trees are frequently colonized by opportunistic and saprophytic fungi such as *Aspergillus*, *Fusarium*, and *Trichoderma*, which participate in wood degradation and may alter the ecological interactions among the host tree, nematode, and insect vector (Vicente et al., 2021). Inácio et al. (2015) characterized the mycobiota associated with *Monochamus galloprovincialis* in Portugal and recovered filamentous fungi belonging to 18 genera, including *Acremonium*, *Alternaria*, *Arthrinium*, *Aspergillus*, *Beauveria*, *Bipolaris*, *Botryosphaeria*, *Botrytis*, *Cladosporium*, *Clonostachys*, *Epicoccum*, *Fusarium*, *Ophiostoma* s.l., *Paecilomyces*, *Penicillium*, *Stemphylium*, *Trichoderma*, and *Trichothecium*.

These observations support the concept that pine wilt disease represents a complex pathosystem involving multiple microbial partners rather than a simple nematode-induced disorder.

### 1.4 Current knowledge of conifer diseases in Kyrgyzstan

Compared with the extensive body of research on pine wilt disease in East Asia and Europe, information on fungal diseases of coniferous forests in Kyrgyzstan remains limited. Most available studies have focused on diseases of Schrenk spruce (*P. schrenkiana* Fisch. & C.A. Mey.), particularly in the Issyk-Kul region, where pioneering investigations were conducted by Elchibaev (1958, 1959), Domashov (1960); Zhuravlev et al. (1969), Bilder (2001), and, more recently, Mosolova (2016).

Among the most important needle diseases reported is spruce needle rust caused by *Chrysomyxa deformans*, which infects young shoots and developing needles, producing characteristic orange-yellow uredinial and telial spore masses and often causing deformation of current-year shoots (Elchibaev, 1959). Another widespread foliar disease is spruce needle cast (shute), caused by *Lophodermium macrosporium* (Hart.) Rehm. This pathogen induces chlorosis, needle necrosis, and premature needle shedding, primarily affecting one-year-old needles on the lower branches of young trees (Zhuravlev et al., 1969).

Despite these valuable early investigations, knowledge of fungal diversity associated with declining coniferous forests in Kyrgyzstan remains fragmentary. Most historical studies relied primarily on morphological identification of fruiting bodies and herbarium specimens, supported by conventional taxonomic keys. Consequently, little information is available regarding endophytic, saprotrophic, or pathogenic fungal communities inhabiting declining conifers, and virtually no studies have employed modern molecular approaches to characterize the mycobiome of Schrenk spruce. This lack of information highlights the need for integrated surveys that combine classical mycology with DNA-based identification methods to improve understanding of fungal diversity and its role in the health and decline of Kyrgyzstan’s mountain forests.

Despite the increasing decline of Schrenk’s spruce forests in the Western Tien Shan, the fungal communities associated with diseased trees remain largely unexplored. The present study aimed to characterize the mycobiota associated with symptomatic *P. schrenkiana* in the Sary-Chelek Biosphere Reserve using morphological and molecular identification, to evaluate fungal diversity and to identify potential pathogenic species contributing to forest decline. The findings provide the first comprehensive assessment of fungal communities associated with diseased Schrenk’s spruce in Kyrgyzstan and establish an important baseline for future forest health monitoring and conservation programs.

## 2 Materials and methods

### 2.1 Study area

The study was conducted in the Sary-Chelek Biosphere Reserve, located in the western Tian Shan Mountains of Kyrgyzstan (41°52′N, 71°59′E). The reserve occupies an altitudinal range from approximately 1, 200 to over 3, 500 m above sea level and encompasses a mosaic of mountain forests, subalpine meadows, river valleys, and alpine ecosystems. Spanning 23, 868 hectares along the eastern slope of the Chatkal Ridge, the reserve encompasses a range of landscapes—from foothill steppes to snow-capped peaks and swift rivers. It supports more than 1, 000 plant species, including around 150 varieties of trees and shrubs, with rare species such as walnut and Regel pear. Sary-Chelek is recognised for having the highest biodiversity in Central Asia, accounting for about 30% of Kyrgyzstan’s total biodiversity, with over 800 species of grasses and 113 tree and shrub species (UNEP-WCMC, 2026). Schrenk spruce (*Picea schrenkiana* Fisch. & C.A. Mey.) forms the dominant coniferous forests at middle elevations, where it plays a key ecological role in maintaining watershed stability and biodiversity. Schrenk’s spruce, also known as *Picea tianschanica*) is a tall, slender conifer that typically reaches 35–45 metres, with exceptional specimens growing up to 60 metres. The trunk can reach up to 2 metres in diameter. This species is characterised by its narrow, pyramidal crown—often pointed or nearly cylindrical—and branches that extend close to the ground, gently drooping at the tips (Sary-Chelek Reserve, 2004).

Soil cover shows pronounced altitudinal zonation typical of the western Tian Shan Mountains. Light and dark sierozems occur in the lower foothills, while mountain brown forest soils dominate the forest belt supporting walnut-fruit forests and Schrenk spruce stands. The physicochemical properties of brown forest soils that support pine tree growth are characterized by a slightly acidic to acidic pH. The pH of the aqueous extract (pH H_2_O) typically ranges from 4.5 to 6.5. In the upper accumulative horizon, humus content varies between 6- 10%. The humus composition displays a pronounced fulvic or humate-fulvic character. Within the humus horizon, the soil structure is lumpy, granular, or finely nutty, and is notably stable due to the binding of humus with iron (Mamytov, 1996).

### 2.2 The climate at the research site

The reserve’s territory is shielded from cold air masses during winter, resulting in relatively mild winters, minimal snowfall, and warm, humid summers. The average temperature in the coldest month, January, is -4.9 °C, with frost recorded at -27 °C. Conversely, the average temperature in the warmest month, June, is 21.9 °C, with peak temperatures reaching up to 38 °C in July. Rainfall averages 836 mm, with about 42% falling in spring, 30% in winter, roughly 20% in summer, and up to 8% in autumn.

### 2.3 Field survey and sample collection

Surveys were performed under stable weather conditions, with an average daytime air temperature of approximately 20 °C. Field sampling was conducted in October 2021–2022 at the selected study sites within the Sary-Chelek Biosphere Reserve. Sampling focused on natural stands of Schrenk spruce (*P. schrenkiana*) trees. Trees were inspected for external symptoms, including crown dieback, needle discolouration, resin exudation, bark necrosis, stem cankers, and wood decay. Samples of symptomatic tissues—including needles, shoots, branches, bark, wood, cones, and fruiting bodies of fungi—were collected using sterile tools, individually labelled, and transported to the laboratory for mycological examination. **Table 1** summarizes the geographic locations and descriptions of the sampling sites. At each site, we selected three symptomatic trees for detailed examination. From each tree, three branches exhibiting visible symptoms of decline or disease were collected. One representative needle was sampled from each selected branch for mycological analysis. Samples included roots, branches, bark, and needles collected from trees showing symptoms of decline, including needle chlorosis, yellowing, reddish-brown discoloration, crown dieback, and wood deterioration (**Figure 3**). Whenever fungal fruiting bodies were present, they were also collected for subsequent identification.

**Table 1:** Data from selected natural samples during the expedition.

№	Sample name	Altitude and GPS data
1	Roots of diseased trees - Picea schrenkiana subsp. tianschanica	1920 m N=41.915839 E=71.929957
2	Schrenk spruce bark with signs of disease	1920 m N=41.915839 E=71.929957
3	Branches of diseased trees - Picea schrenkiana subsp. tianschanica	1920 m N=41.915839 E=71.929957
4	Fir needles of Picea schrenkiana subsp. tianschanica with signs of disease	1892 m N=42.873715 E=71.97.8464
5	The soil under the Tien Shan Spruce *Picea schrenkiana subsp. tianschanica* (10-15 cm)	1854 m N=41.866469 E=71.976131
6	Juniper Virginia Dendrosad	1264 m N=41.808923 E=71.956775
7	The soil under the juniper	1400 m N=41.798014 E=71.935250
8	Bark of Tien Shan Spruce *Picea schrenkiana subsp. tianschanica* ( 150-200 years old)	1461 m N=41.79.7530 E=71.930009
9	Fir needles of Picea schrenkiana subsp. tianschanica	1450 m N=41.797644 E=71.930329
10	Dry rot of the branches of Picea schrenkiana subsp. tianschanica	1450 m N=41.797488 E=71.930038

**Figure 3:**
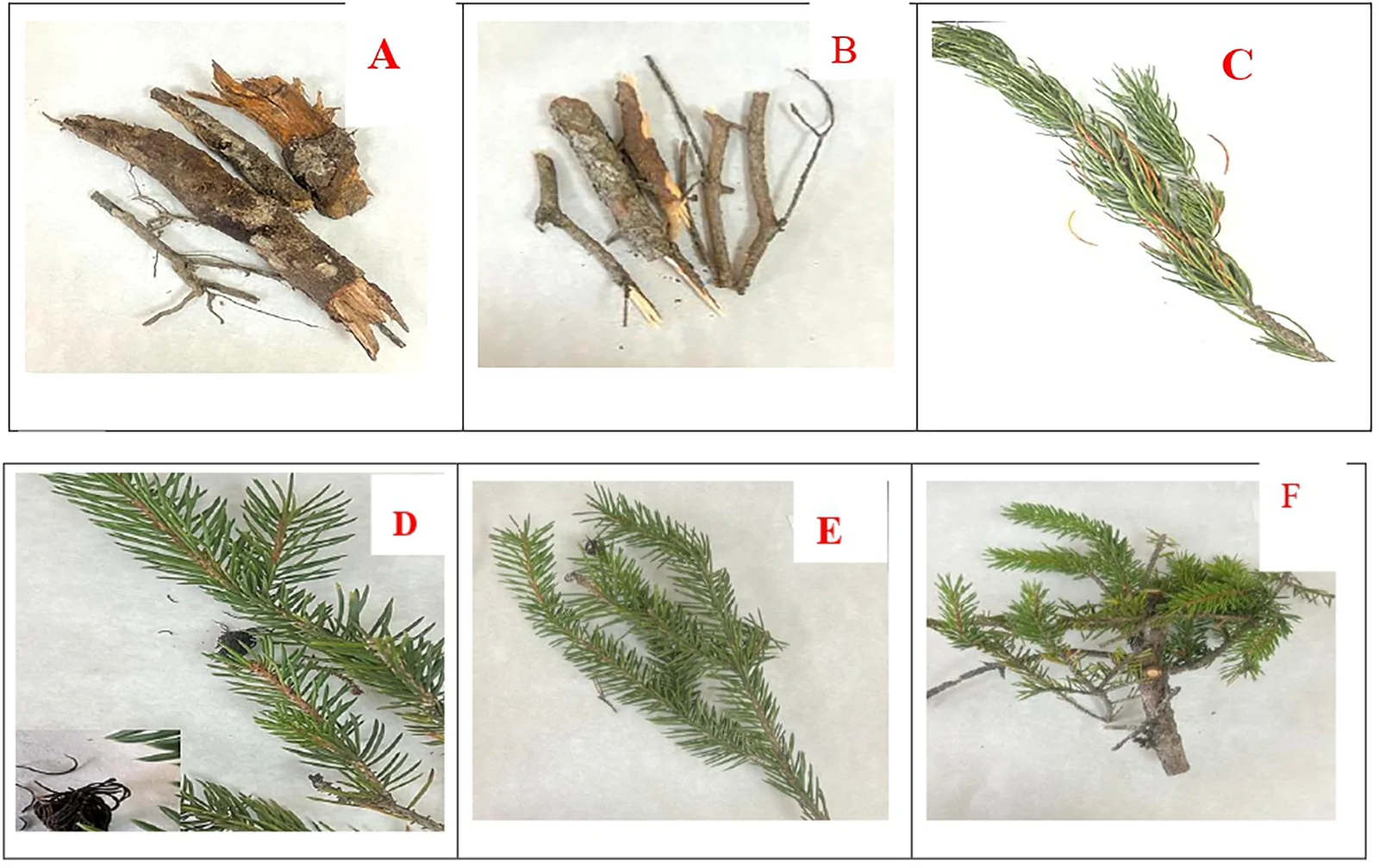
Representative samples were collected from symptomatic tissues of coniferous trees as follows: **(A)** and **(B)** are segments from the symptomatic roots of diseased spruce; **(C–E)** are needle segments from diseased spruce; and **(F)** is a stem segment from diseased spruce.

All samples were placed individually in sterile paper bags, labelled with a unique sample code, and transported to the laboratory for fungal isolation and identification.

To minimize contamination, sampling tools were disinfected with 70% ethanol between collections. Following transport, symptomatic tissues were processed within 24 h.

### 2.4 Isolation and preservation of fungal isolates

Symptomatic plant tissues were cut into 5–10 mm segments from the interface between healthy and diseased tissue. The segments were surface-sterilized in 2.5% sodium hypochlorite solution for 3–5 min following the protocol of Guo et al. (2008), rinsed three times with sterile distilled water, air-dried on sterile filter paper, and placed onto 2% water agar (WA) plates. Plates were incubated at 25 ± 1 °C until fungal hyphae emerged from the plant tissues, typically within 5–7 days. Hyphal tips were aseptically transferred to Potato Dextrose Agar (PDA) to obtain pure cultures. Additional subcultures were established on Czapek Dox Agar (CDA) and Synthetic Nutrient-poor Agar (SNA) to promote the development of diagnostic morphological characteristics. Pure fungal isolates obtained from needles, branches, roots, bark, and other symptomatic tissues were maintained on PDA and preserved at −80 °C in 20% glycerol for long-term storage.

### 2.5 Morphological identification of fungal isolates

Morphological identification was performed using cultures grown on PDA, CDA, and SNA media. Colony characteristics, including growth rate, texture, pigmentation, margin morphology, aerial mycelium, and sporulation patterns, were recorded after incubation (Barnett and Hunter, 1998; Carrillo, 2003). Microscopic observations focused on hyphal, conidiophore, conidiogenous cell, macroconidial, microconidial, and other reproductive structures.

Microscopic preparations were mounted in lactophenol cotton blue and examined using a compound light microscope. Digital images were captured with Motic Images Plus software (version 2.0 ML). For each isolate, the length and width of 30 randomly selected conidia were measured using an ocular micrometer. Fungal isolates were identified based on their cultural and microscopic characteristics using standard taxonomic references, including Illustrated Genera of Imperfect Fungi (Barnett and Hunter, 1998), Illustrated Dictionary of the Fungi (Alexopoulos et al.1996), Pictorial Atlas of Soil and Seed Fungi (Watanabe, 2010), and the Simplified Key to the Identification of Common Fungi (Williams-Woodward, 2001). Species identification was based on the combination of colony morphology, reproductive structures, and morphometric characteristics. We employed strains from our curated collection of *Penicillium, Fusarium*, and *Trichoderma* species as experimental controls to ensure reliable benchmarking.

### 2.6 DNA extraction of fungal isolates

Fungal samples were cultured on Potato Dextrose Agar (PDA) and Yeast Extract Agar (YEA) at 25 °C for DNA extraction. Approximately 100 mg of mycelial material was transferred to a screw-cap 2 mL microcentrifuge tube containing Lysing Matrix C. Genomic DNA was extracted using the DNAs Plant Mini Kit (Qiagen) according to the manufacturer’s instructions, with the following modifications: samples were homogenized in 400 µL of AP1 buffer for 60 seconds at 6.0 m/s using a FastPrep-24 instrument (MP Biomedicals), followed by a brief centrifugation to pellet debris. RNase A (4 µL of 100 mg/mL) was added to the lysate, and the mixture was incubated at 65 °C for 10 minutes. Purified DNA was eluted in 100 µL of buffer AE and stored at –20 °C until further use. DNA was amplified from fungal samples using standard Taq DNA polymerase (New England Biolabs). Amplification targeted the complete ribosomal RNA (rrn) operon internal transcribed spacer (ITS) region, encompassing ITS1, ITS2, ITS4 and the 5.8S rRNA gene with the primers: TCCGTAGGTGAACCTGCGG/TCCTCCGCTTATTGATATGC.

Species-level identification was achieved using specific molecular markers: Translation longation Factor 1-alpha (TEF1-α): EF1-728F/EF1-986R, CATCGAGAAGTTCGAGAAGG/TACTTGAAGGAACCCTTACC.

Thermal cycling conditions were as follows: initial denaturation at 95 °C for 2 min, followed by 35 cycles of denaturation at 95 °C for 45 s, annealing at the primer-specific temperature for 45 s, and extension at 68 °C, with a final extension at 68 °C for 5 min. We verified PCR product sizes by agarose gel electrophoresis and purified products using the QIAquick PCR Purification Kit (Qiagen). PCR products were subjected to Sanger sequencing at Macrogen (Seoul, Korea). Consensus sequences for each marker were assembled from the raw reads using MEGA6. The consensus sequences were queried against the GenBank nucleotide database using BLASTn to identify closely related entries. From the top 100 hits ranked by similarity, sequences with query coverage of at least 80% and assigned to fungal genera were retained for the reference dataset. Phylogenetic trees were reconstructed using the Neighbour-Joining (NJ) algorithm with p-distance as the substitution model in MEGA6. Topological robustness was assessed by nonparametric bootstrap analysis with 1, 000 replicates. Phylogenetic trees were constructed as unrooted, with no outgroup included to avoid bias in branching patterns.

### 2.7 Statistical analysis

The Shannon index was used to determine the complete species composition of the fungal communities, including both abundant and rare species, present at the studied sites (Statology, 2021). The Shannon–Weaver diversity index is based on information theory. The Shannon function H′ measures uncertainty, corresponds to the entropy concept, and is calculated using the following equation: H′ = −Σ(pi ln pi), (1) where pi = ni/N (the share of the i-species in the biotope), ni = the number of the i-species (specimen), N = the total number of microbial species, ln = the natural logarithm, Σ is the Greek symbol that means “sum, “ and p = the proportion of the entire community made up of species I. The higher the value of H′, the higher the species diversity in a particular community. The lower the value of H′, the lower the diversity. A value of H′ = 0 indicates a community with only one species. The obtained data were statistically processed using SPSS version 25 software (IBM, USA).

## 3 Results

### 3.1 Identification of the primary factors associated with the decline and mortality of Schrenk’s spruce

Field surveys revealed progressive crown dieback characterized by complete needle loss, bark deterioration, and extensive tree mortality. In the lower stem section (up to approximately 0.5 m above ground level), the bark remained moist and firmly attached to the trunk. In contrast, above 2 m, the bark was severely desiccated and detached easily, exposing the underlying sapwood. Removal of the bark revealed extensive galleries and feeding scars typical of longhorn beetle (*Coleoptera: Cerambycidae*) infestation. Numerous larval tunnels and frass deposits were observed beneath the bark, and living beetle larvae were recovered from affected trees (**Figure 4A**). The wood exhibited reduced mechanical resistance, longitudinal cracking, and multiple emergence holes, indicating advanced deterioration of xylem tissues associated with insect colonization.

**Figure 4:**
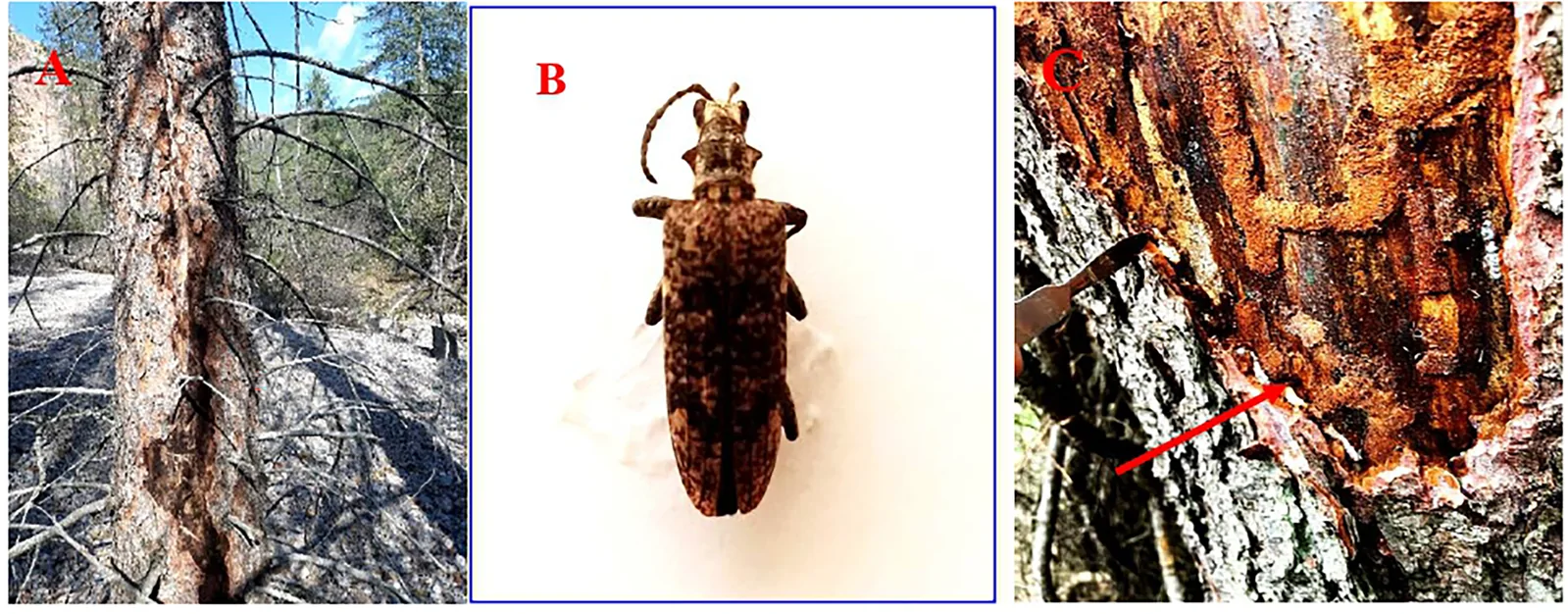
**(A)** A heavily affected Tien Shan Spruce tree; **(B)** Pine bronze longhorn beetle (*Monochamus galloprovincialis Olivier*); **(C)** Pest larvae located under the bark of the affected tree.

The field survey indicated a relatively low overall density of insect infestations within the investigated forest stands. Collected specimens included representatives of the families *Scolytidae* (bark beetles), *Buprestidae* (jewel beetles), and *Cerambycidae* (longhorn beetles). Infested trees were consistently characterized by poor physiological condition and exhibited extensive root damage.

Morphological examination of the collected adult specimens identified all longhorn beetles as female *Monochamus galloprovincialis* (Cerambycidae) (**Figures 4B, C**). In contrast, the immature stages (larvae) of *Scolytidae* and *Buprestidae* could not be identified to species level because of the limited availability of diagnostic morphological characters. To determine whether pine wilt disease was involved in the observed decline, wood samples from symptomatic trees were examined for the presence of the pine wood nematode (*Bursaphelenchus xylophilus*). No nematodes were detected in any of the analyzed samples.

### 3.2 Fungal species isolated from symptomatic Schrenk’s spruce needles

Mycological analysis of symptomatic Schrenk’s spruce (*P. schrenkiana*) needles revealed a diverse fungal community dominated by species of *Penicillium* and *Alternaria*. We recovered multiple fungal isolates from individual needle samples, with numbers ranging from 3 to 10 per sample.

Of the 52 isolates obtained, *Penicillium* spp. accounted for approximately 50% of the fungal community, whereas *Alternaria* spp. accounted for nearly 30%. The remaining isolates consisted of *Curvularia* spp. (approximately 10%) and *Didymella* spp. (approximately 10%).

#### 3.2.1 Penicillium philippinense

Colonies identified as *Penicillium philippinense* exhibited rapid growth on Czapek agar, producing compact, velvety to floccose colonies with a reddish-brown surface (**Figure 5С**). The colony surface developed irregular greyish-red furrows, whereas the reverse side appeared pale yellowish-brown with slight yellow pigmentation diffusing into the surrounding medium. Conidial structures were sparse and produced transparent exudate droplets. Microscopic examination revealed thick-walled, septate hyphae composed of polygonal cells. Conidiophores arose from aerial hyphae and measured predominantly 30–127 (200) × 2.5–3.5 (–3.7) μm. Their walls were finely roughened, septate, and terminated in slightly swollen apices. Metulae were arranged predominantly in pairs or groups of three and measured 14–20 × 2.3–3.2 μm. Phialides occurred in clusters of four to seven, forming predominantly biverticillate, asymmetrical conidiophores. Conidia were ovoid, smooth-walled, measuring 2.2–3.0 × 2.0–2.7 μm, and formed long, tangled chains exceeding 100 μm in length (**Figure 5D**). ITS sequence analysis confirmed the morphological identification of isolate Sch-1 as *Penicillium philippinense*, forming a well-supported clade with reference sequences retrieved from GenBank (**Figure 5Е**).

**Figure 5:**
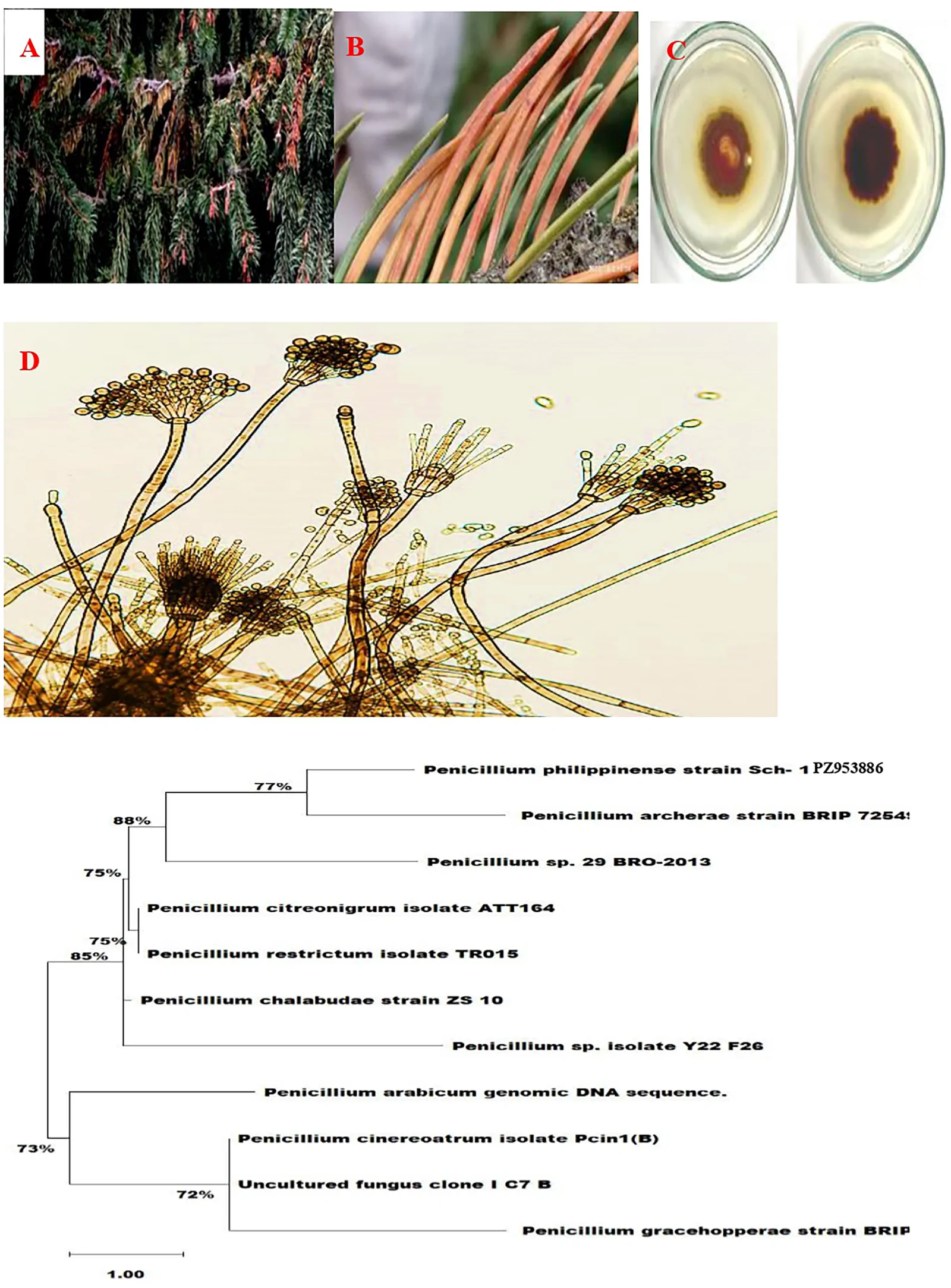
Morphological and molecular characterization of *Penicillium philippinense* isolated from symptomatic Schrenk's spruce (*Picea schrenkiana*) needles: **(A, B)** Diseased needles exhibiting fungal infection symptoms; **(C)** colony morphology on potato dextrose agar (PDA); **(D)** microscopic features showing mycelia, conidiophores, and conidia. Scale bars: D = 100 μm. **(E)** Neighbour-joining tree of the ITS1-5.8S-ITS2 rDNA region, showing the position of isolate *Penicillium philippinense* Sch-1 described in this paper and other closely related *Penicillium* species. The phylogenetic tree is not rooted.

#### 3.2.2 Alternaria alternata

Representative symptoms and morphological characteristics of *Alternaria alternata* isolated from symptomatic Schrenk’s spruce needles are presented in **Figure 6**. Colonies developed rapidly on potato dextrose agar (PDA) and exhibited a dense, velvety to felted texture. Initially pale grey, the colonies gradually darkened with age, becoming olive-black at maturity. Mature cultures showed distinct concentric zonation, corresponding to the characteristic ring-like growth pattern (**Figure 6B**).

**Figure 6:**
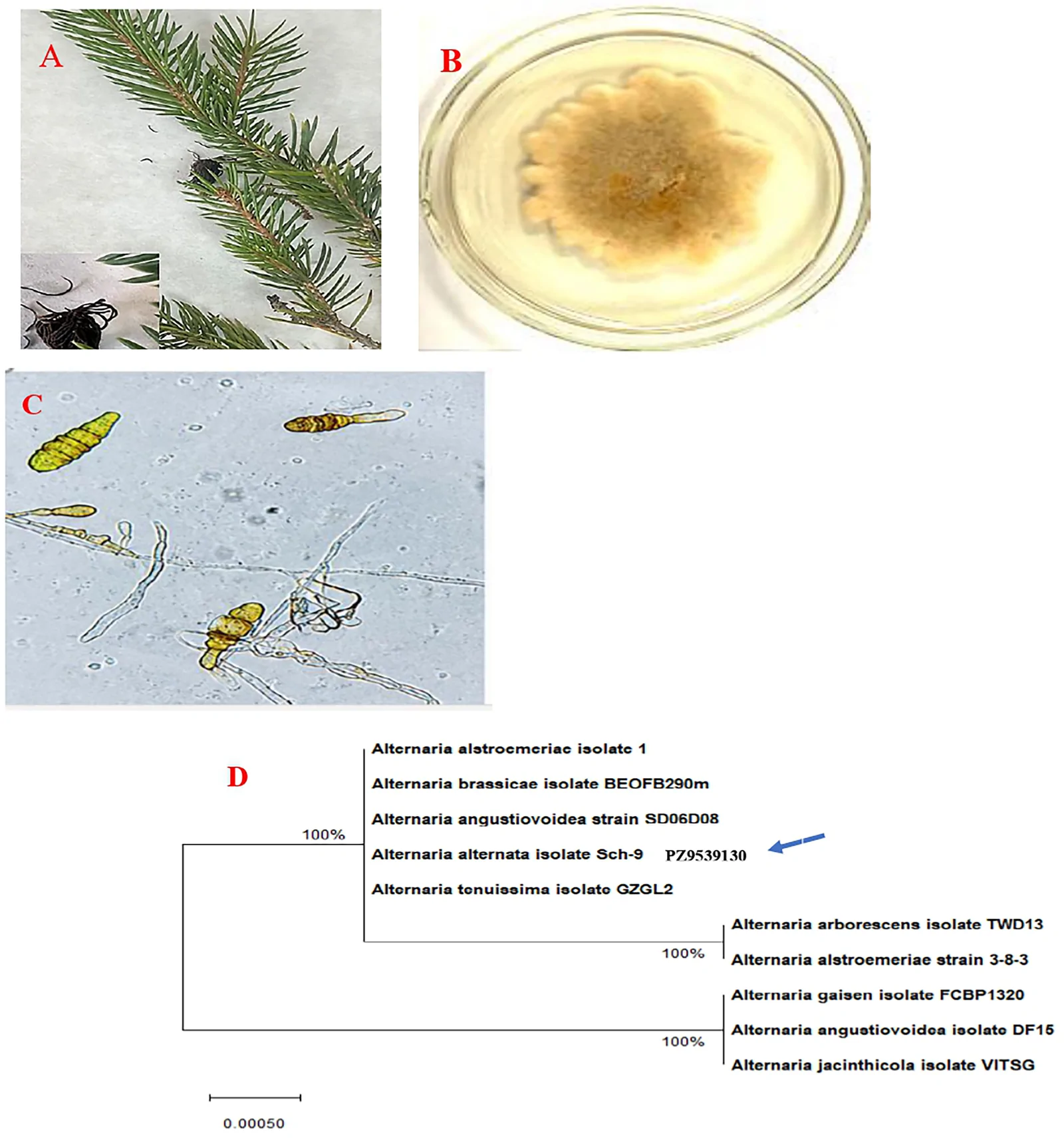
Morphological and molecular characterization of *Alternaria alternata* isolated from symptomatic Schrenk's spruce (*Picea schrenkiana*) needles. **(A)** Diseased needles exhibiting fungal infection symptoms; **(B)** colony morphology on potato dextrose agar (PDA); **(C)** microscopic features showing mycelia, conidiophores, and conidia. Scale bars: C = 50 μm; **(D)** Neighbour-joining tree of the ITS1-5.8S-ITS2 rDNA region, showing the position of isolate *Alternaria alternata* Sch-9 described in this paper and other closely related Alternaria species. The phylogenetic tree is not rooted.

Microscopic examination revealed abundant conidia that were obclavate to club-shaped, pale to golden brown, measuring 20–32 × 7–10 μm. Conidia possessed 2–4 transverse septa together with several longitudinal or oblique septa and gradually tapered into a distinct cylindrical beak. Conidiophores were short, septate, simple or occasionally branched, and frequently exhibited a characteristic geniculate (zigzag) morphology (**Figure 6C**). ITS sequence analysis confirmed the morphological identification of isolate Sch-9 as *Alternaria alternata*, forming a well-supported clade with reference sequences retrieved from GenBank (**Figure 6D**).

#### 3.2.3 Curvularia rouhanii

Colonies of *Curvularia rouhanii* developed slowly on potato dextrose agar (PDA). Young cultures were pale grey and gradually became orange to brownish-orange with age. Mature colonies exhibited a cottony to floccose texture and developed characteristic lobed, undulate margins (**Figure 7B**).

**Figure 7:**
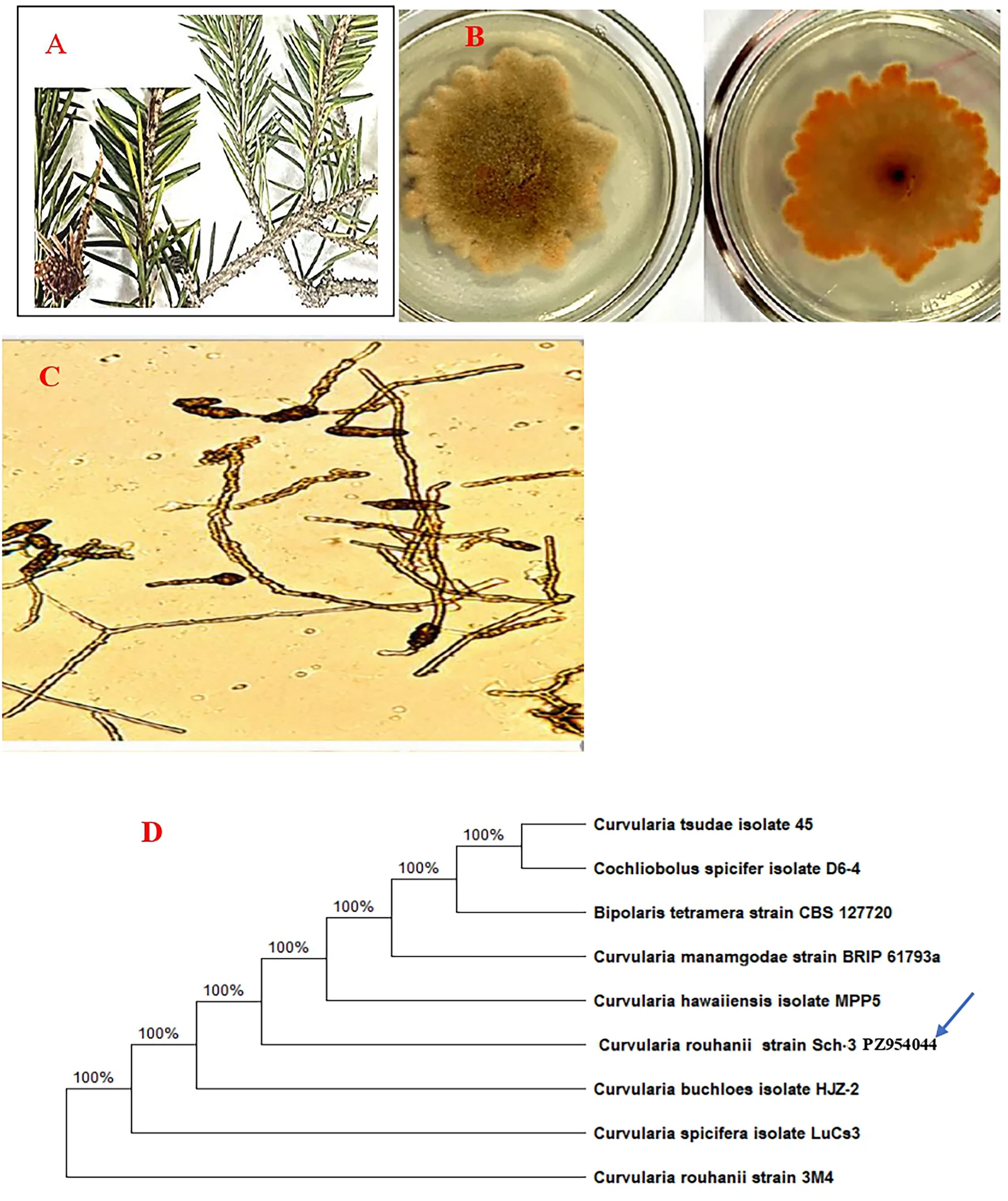
Morphological and molecular characterisation of *Curvularia rouhanii* isolated from symptomatic Schrenk's spruce (*Picea schrenkiana*) needles. **(A)** Diseased needles exhibiting fungal infection symptoms; **(B)** colony morphology on potato dextrose agar (PDA); **(C)** microscopic features showing mycelia, conidiophores, and conidia. Scale bars: C = 100 μm; **(D)** Neighbour-joining tree of the ITS1-5.8S-ITS2 rDNA region, showing the position of isolate *Curvularia rouhanii* Sch-3 described in this paper and other closely related *Curvularia* species. The phylogenetic tree is not rooted.

Microscopic examination revealed branched, septate hyphae that were pale brown, smooth to finely verrucose-walled, and 1–4 μm wide. Conidiophores were erect to slightly curved, occasionally branched, with smooth to finely verrucose walls, measuring 43–103 × 3–5 μm. Conidiogenous cells were terminal or subterminal, pale brown with conspicuous darkened conidial scars, subcylindrical to slightly swollen, and 3–5 μm wide. Conidia were predominantly 2–3-septate, straight to slightly curved, with a mean size of 8.2 (± 3.32 × 3.3 (± 0.65) μm. The third median cell was consistently larger than the remaining cells, whereas the apical and intermediate cells possessed smooth walls. The basal cell measured 13.0 (± 3.19) × 6.71 (± 1.15) μm (**Figure 7C**). ITS sequence analysis confirmed morphological identification, placing the isolate within the *Curvularia rouhanii* clade with strong bootstrap support (**Figure 7D**).

#### 3.2.4 Didymella glomerata

Representative morphological characteristics of *Didymella glomerata* isolated from symptomatic Schrenk’s spruce (*P.schrenkiana*) tissues are shown in **Figure 8**.

**Figure 8:**
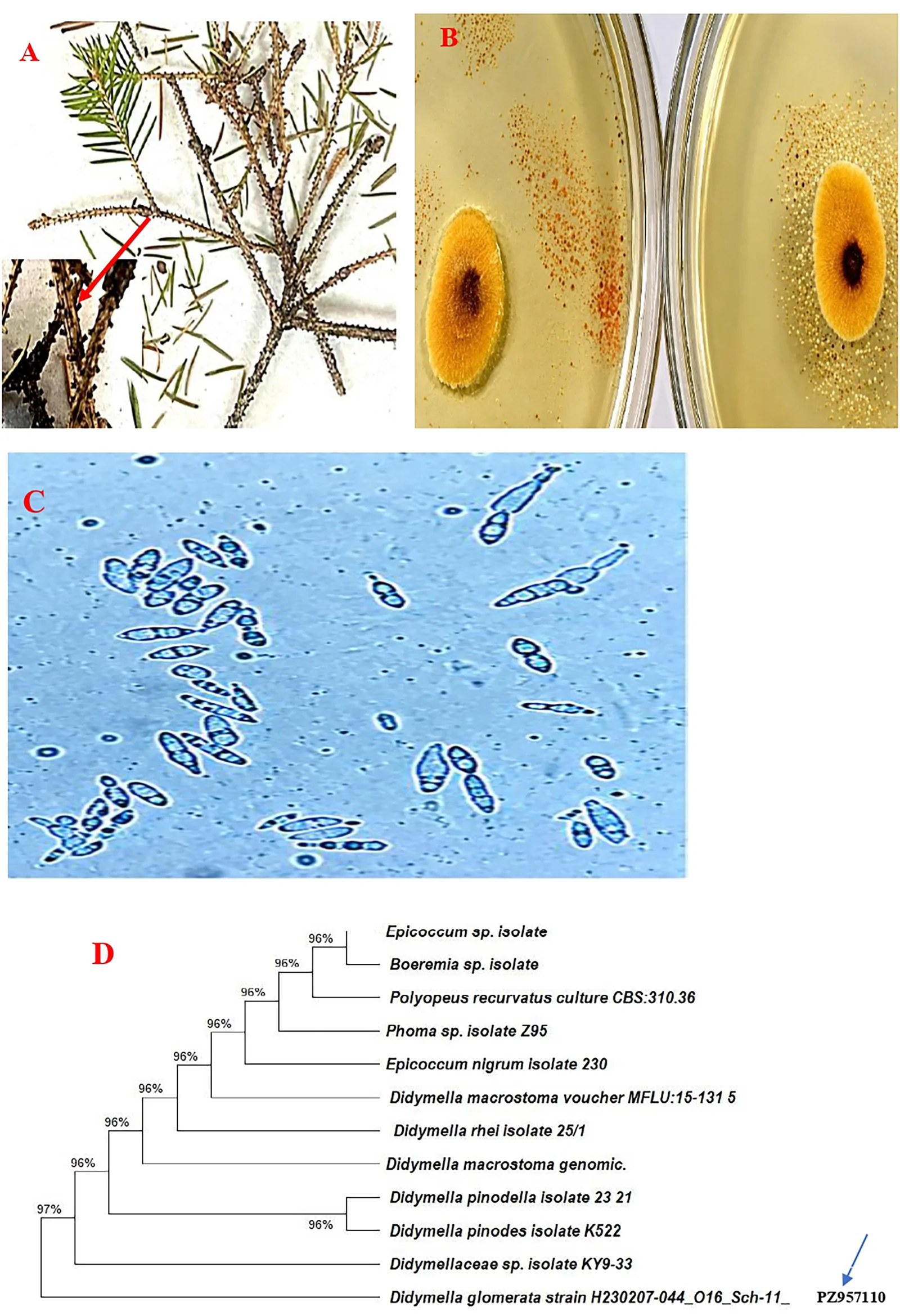
Morphological characterisation of *Didymella glomerata* isolated from symptomatic Schrenk's spruce (*Picea schrenkiana*). **(A)** Symptoms observed on infected needles and shoots; **(B)** colony morphology on potato dextrose agar (PDA); **(C)** conidiogenous cells; hyaline aseptate conidia. Scale bars: C = 50 μm; **(D)** Neighbour-joining tree of the ITS1-5.8S-ITS2 rDNA region, showing the position of isolate *Didymella glomerata*, Sch-11 (accession number is PZ957110) described in this paper and other closely related *Didymella* species. The phylogenetic tree is not rooted.

Dark, globose pycnidia developed on infected needles and small branches collected from diseased trees (**Figure 8A**). Microscopic examination revealed numerous smooth-walled, ampulliform to subglobose conidiogenous cells measuring 3.3–7.0 × 3.0–7.0 μm. Conidia were predominantly oblong to cylindrical, occasionally ovoid or obovoid, hyaline, aseptate, and smooth- to thin-walled. They measured 4.3–6.1 × 1.0–3.0 μm, with a mean size of 5.7 ± 0.4 × 2.3 ± 0.2 μm (**Figure 8C**).

ITS sequence analysis confirmed morphological identification, placing the isolate Sch-11 within the *Didymella glomerate*, clade with strong bootstrap support (**Figure 8D**).

### 3.3 Fungal species isolated from diseased roots of Schrenk’s spruce

Mycological analysis of diseased roots collected from symptomatic Schrenk’s spruce (*P. schrenkiana*) revealed a fungal community dominated by species of the genera *Penicillium* and *Talaromyces*. Among the recovered isolates, *Penicillium* showed the highest species diversity and included *Penicillium montanense*, *Penicillium velutinum*, *Penicillium cinereoatrum* and *Penicillium philippinense*. In contrast, *Talaromyces* was recovered less frequently and was represented by fewer isolates.

The repeated isolation of multiple *Penicillium* species from diseased root tissues indicates that members of this genus constitute an important component of the root-associated fungal community in declining Schrenk’s spruce. **Figures 9**, **10** present representative isolates and their morphological characteristics.

**Figure 9:**
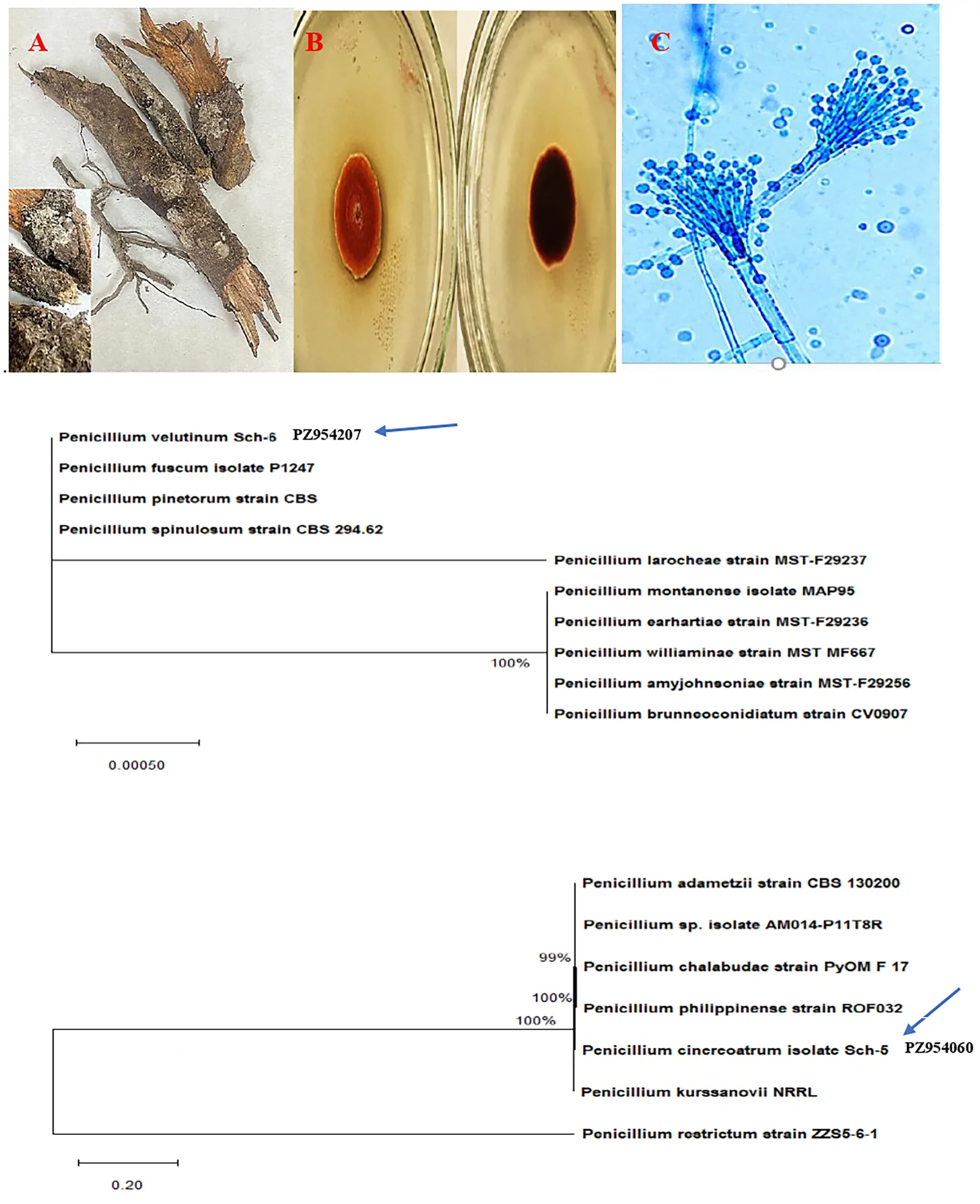
Representative fungal species isolated from diseased roots of Schrenk's spruce (*Picea schrenkiana*). **(A)** root segments of Schrenk spruce displaying symptoms of fungal infection; **(B)** Colony morphology on potato dextrose agar (PDA); **(C)** microscopic characteristics: conidiophores, and conidia. Scale bars: C = 100 μm; **(D, E)** Neighbour-joining tree of the ITS1-5.8S-ITS2 rDNA region, showing the position of isolates Sch-6 as *Penicillium velutinum*, and Sch-5 as *Penicillium cinereonotum*, described in this paper and other closely related *Penicillium* species. The phylogenetic tree is not rooted.

**Figure 10:**
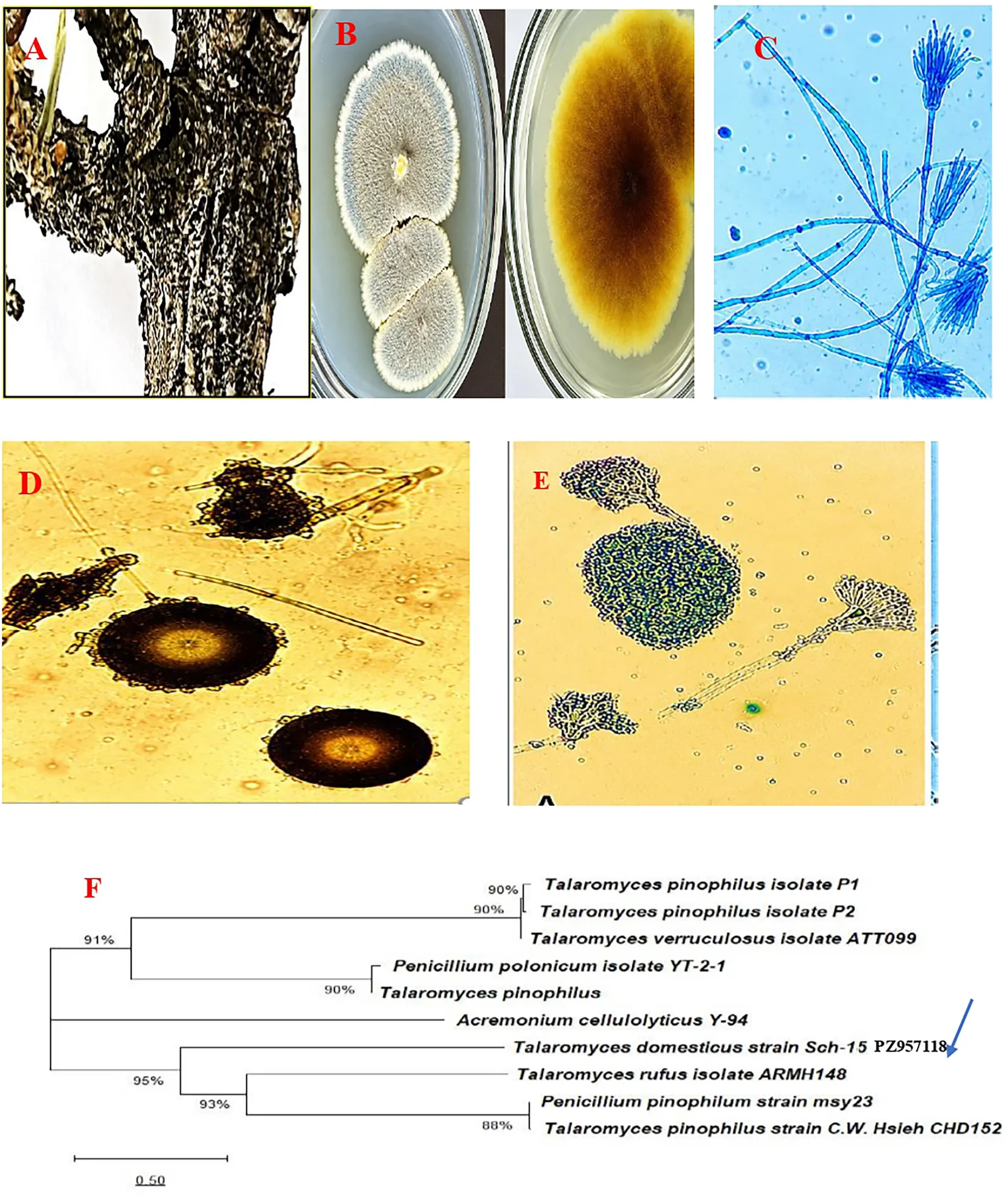
Representative fungal species isolated from diseased roots of Schrenk's spruce (*Picea schrenkiana*). **(A)** Root segments of Schrenk spruce displaying symptoms of fungal infection; **(B)** Colony morphology on potato dextrose agar (PDA); **(C)** microscopic characteristics: conidiophores and conidia; **(D)** conidiophores and ascospores. **(E)** pycnidia and conidiophores. Scale bars: C, D, E = 100 μm; **(F)** Neighbour-joining tree of the ITS1-5.8S-ITS2 rDNA region, showing the position of isolate *Talaromyces domesticus* Sch-15 described in this paper and other closely related *Talaromyces species*. The phylogenetic tree is not rooted.

The colonies of *Talaromyces is*olates on the PDA exhibited textures ranging from flocculose to funiculose and displayed a pale-yellow color with a subtle greenish tint (**Figure 10B**). The reverse side of the colonies was yellowish-brown (**Figure 10C**). Conidiophores measured 150–250 × 2.5–3.5 µm. Metulae were arranged in whorls of 3–5 and measured 12–14.5 × 2.5–4 µm. Phialides were acicular, numbering 3–6 per metula, and measured 12–13.5 × 2.0–3.0 µm. Conidia were smooth, hyaline, and ellipsoidal, measuring 3.0–3.5 × 2.0–2.5 µm (**Figure 10D**). Additionally, the formation of ascospores and pycnidia was observed as the cultures matured on the medium (**Figures 10D, E**). The phylogenetic tree, supported by bootstrap values ranging from 50 to 100, confirms the placement of *Talaromyces domesticus*, Sch-15 within the section *Talaromyces* (**Figure 10F**).

### 3.4 Fungal species isolated from the stem bark of Schrenk’s spruce

Mycological analysis of stem and branch bark collected from symptomatic Schrenk’s spruce (*P. schrenkiana*) revealed that species of the genus *Fusarium* predominated among the recovered isolates. Two species were identified, *Fusarium avenaceum* and *Fusarium acuminatum*, with *Fusarium avenaceum* being the most frequently isolated species (**Figure 11**). Colonies of *F. avenaceum* exhibited loose, fluffy, or cottony aerial mycelium with white to pink or carmine pigmentation at the margins (**Figure 11B**). Lateral conidiophores formed during the initial developmental stages, while secondary conidiophores were flexible and highly branched. Conidiogenous cells were slender and phialidic (**Figure 11E**). Macroconidia produced from sporodochia were uniform, narrowly fusiform, and curved, containing 5–7 septa, an elongated apical cell, and a distinct basal cell. The 5-septate macroconidia measured 63.8 (51.1–80.0) × 4.1 (3.6–4.7) µm, with an apical cell length of 17.1 (14.1–21.2) µm. Occasionally, fusiform conidia with 0–3 septa (10–26 × 2.2–4.2 µm) were present in the aerial mycelium (**Figure 11F**). The phylogenetic tree, supported by bootstrap values ranging from 50 to 100, confirms the placement of *Fusarium avenaceum* Sch-13 within the section *Fusarium* (**Figure 11G**).

**Figure 11:**
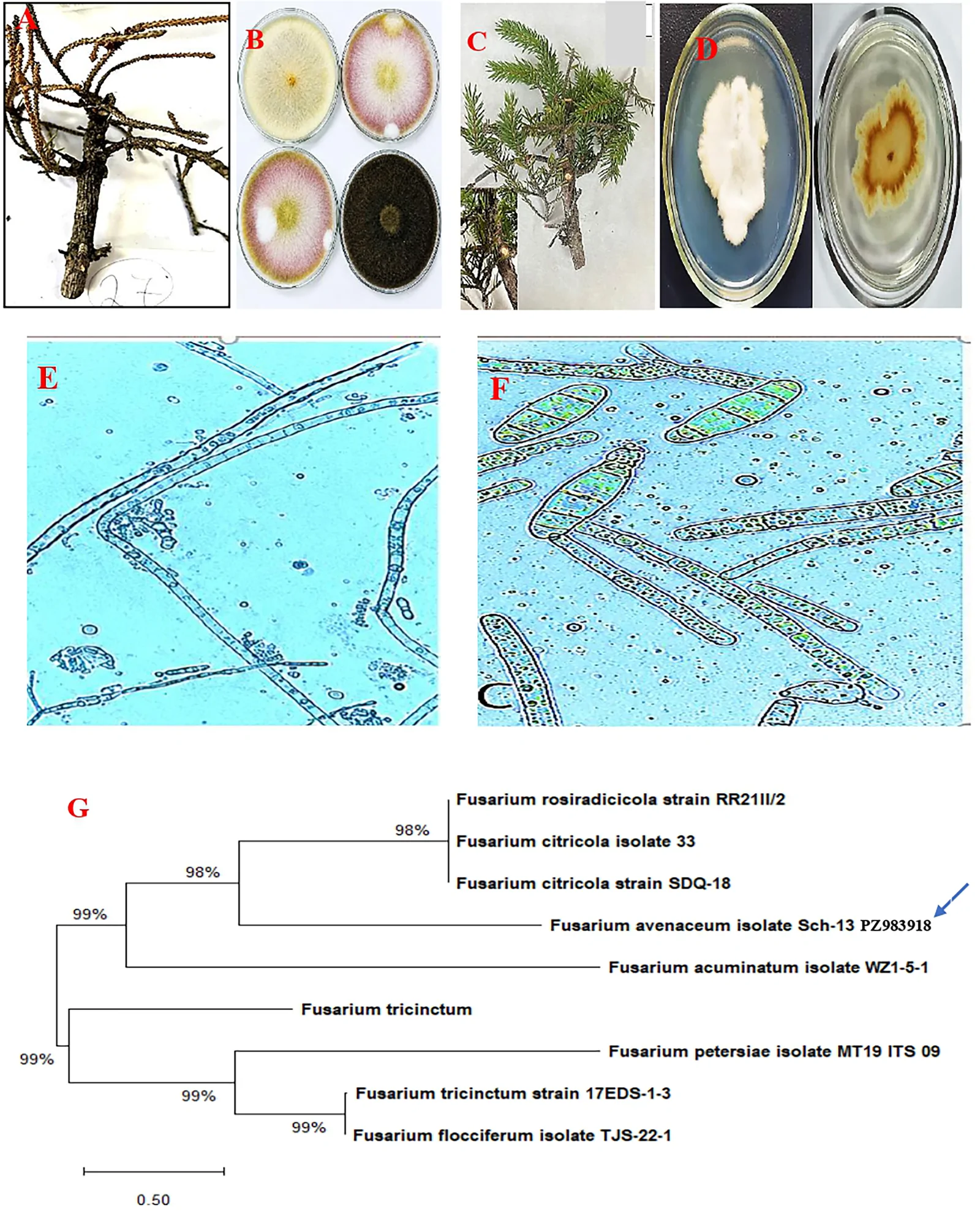
Representative fungal species isolated from diseased stem's bark of Schrenk's spruce (*Picea schrenkiana*). **(A, C)** the stem of Schrenk spruce displaying symptoms of fungal infection; **(B, D)** Colony morphology on potato dextrose agar (PDA); **(E, F)** microscopic characteristics: conidiophores, as well as microconidia and macroconidia of *Fusarium avenaceum* strain Sch-13; Scale bars: E, F = 100 μm; **(G)** Neighbour-joining tree of the ITS1-5.8S-ITS2 rDNA region, showing the position of isolate *Fusarium avenaceum* Sch-13 described in this paper and other closely related *Fusarium species*. The phylogenetic tree is not rooted.

In addition, several isolates belonging to the genus *Trichoderma* were consistently recovered from the same bark samples (**Figure 12**). Cultures exhibited rapid growth on PDA, initially forming white, fluffy colonies. As sporulation progressed, colonies quickly turned dark green, and the surface became powdery with abundant conidia (**Figure 12B**). Conidiophores display irregular branching, often producing verticillate-pyramidal structures. Branches typically arise in pairs or whorls at an angle to the main axis. Phialides are flask-shaped or ampulliform (12C). Conidia are predominantly green, globose to subglobose, and have smooth to slightly roughened walls. Their dimensions generally range from 2.2–2.6 × 1.8–2.5 µm (12D). The phylogenetic tree, supported by bootstrap values ranging from 50 to 100, confirms the placement of *Trichoderma harzianum F-5* within the section *Trichoderma* (**Figure 12E**).

**Figure 12:**
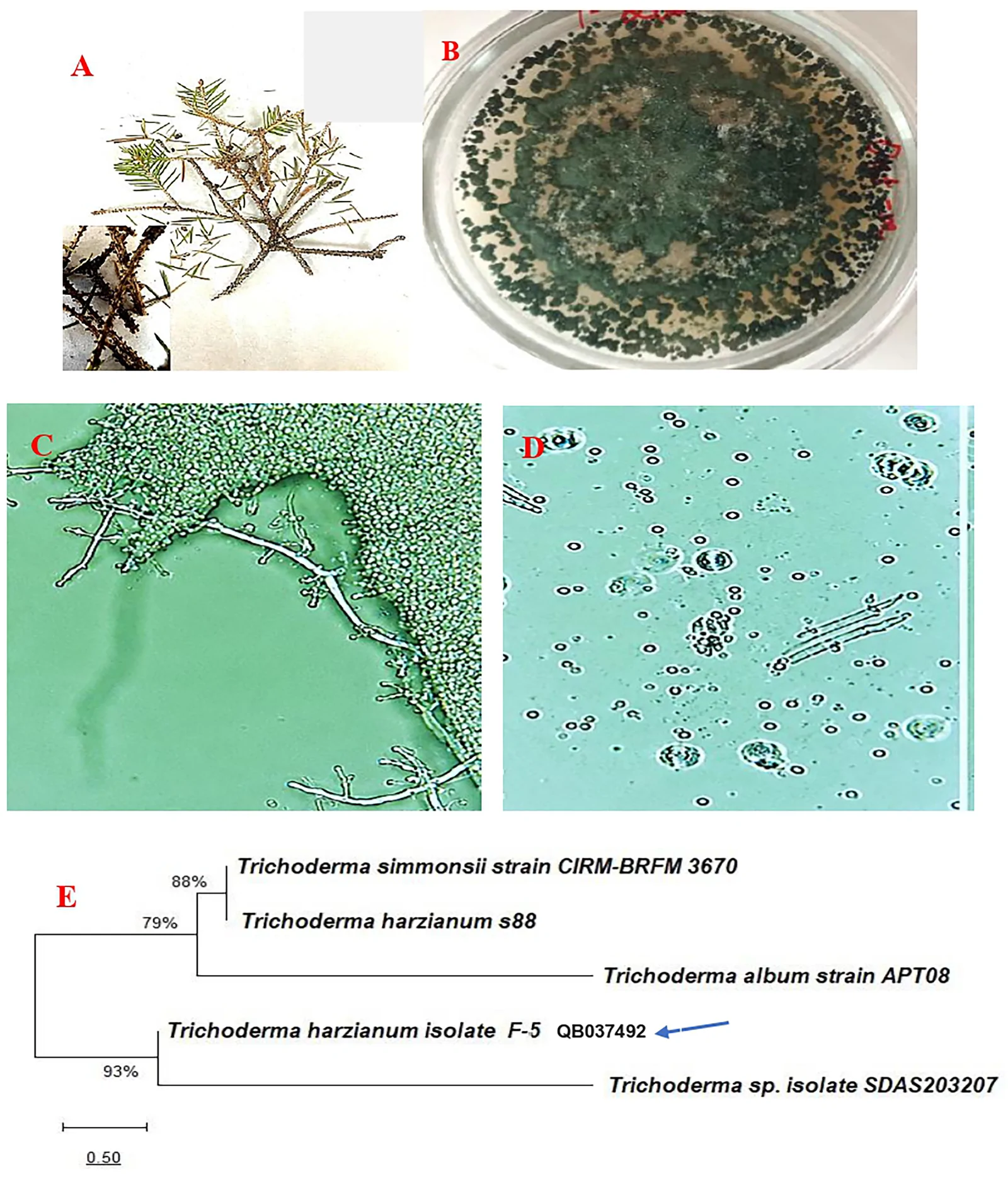
Representative fungal species isolated from the diseased stem bark of Schrenk's spruce (*Picea schrenkiana*). **(A)** the stem of Schrenk spruce displaying symptoms of fungal infection; **(B)** Colony morphology on potato dextrose agar (PDA); **(C, D)** microscopic characteristics: conidiophores, conidia of *Trichoderma harzianum F-5*. Scale bars: C, D = 100 μm; **(E)** Neighbour-joining tree of the ITS1-5.8S-ITS2 rDNA region, showing the position of *Trichoderma harzianum* F-5, described in this paper, and other closely related *Trichoderma species*. The phylogenetic tree is not rooted.

### 3.5 Distribution of fungal communities in different tissues of Schrenk’s spruce

The distribution of fungal taxa varied markedly among the different tissues of symptomatic Schrenk’s spruce (*P.schrenkiana*). Diseased needles harbored the highest fungal diversity, with representatives of four genera identified: *Penicillium*, *Alternaria*, *Curvularia*, and *Didymella*. Among these, *Penicillium* exhibited both the highest species richness and the greatest isolation frequency. In contrast, diseased root tissues yielded a less diverse fungal community, consisting primarily of *Penicillium* species together with isolates of *Talaromyces*. Although closely related to *Penicillium*, *Talaromyces* was recovered less frequently. A total of 52 fungal isolates were obtained from diseased needle samples of Schrenk’s spruce trees. Of these, 25 were identified as *Penicillium*, 15 as *Alternaria*, and six each as *Curvularia* and *Didymella*. From the roots, 32 isolates were recovered, with most (25) belonging to *Penicillium* and the remaining seven to *Talaromyces.* Analysis of bark from diseased trunks yielded 23 fungal isolates, predominantly *Fusarium* (18), with the other five identified as *Trichoderma*.

The fungal community associated with stem bark differed substantially from those of needles and roots. *Fusarium* spp. dominated bark tissues, accounting for approximately 80% of all recovered isolates. *Trichoderma* isolates were also consistently detected in bark samples, where they frequently occurred in association with *Fusarium* spp. Overall, the composition of fungal communities differed among needles, roots, and bark, indicating clear tissue-specific patterns of fungal colonization in declining Schrenk’s spruce (**Figure 13**). These observations indicate that each fungal species occupies a distinct ecological niche within the forest ecosystem. Some species exploit host plant tissues for sustenance and shelter, while others parasitise pathogenic fungi and obtain nutrients from their mycelia.

**Figure 13:**
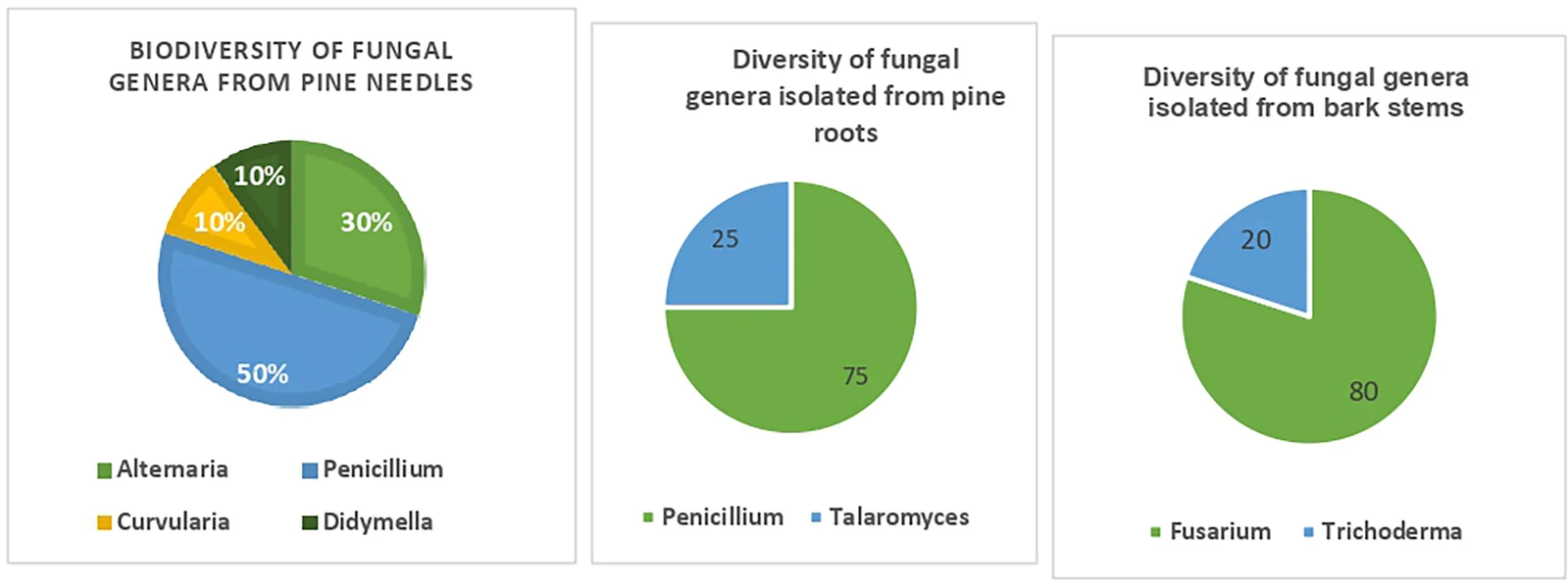
Distribution of fungal genera isolated from diseased Schrenk's spruce in the Sary-Chelek Biosphere Reserve.

The assessment of fungal community biodiversity on Schrenk spruce organs with disease symptoms—measured using the Shannon index—revealed generally low values throughout the study season. Notably, higher index values were observed on the needles of diseased trees, whereas roots and trunks consistently showed low biodiversity (**Figure 14**).

**Figure 14:**
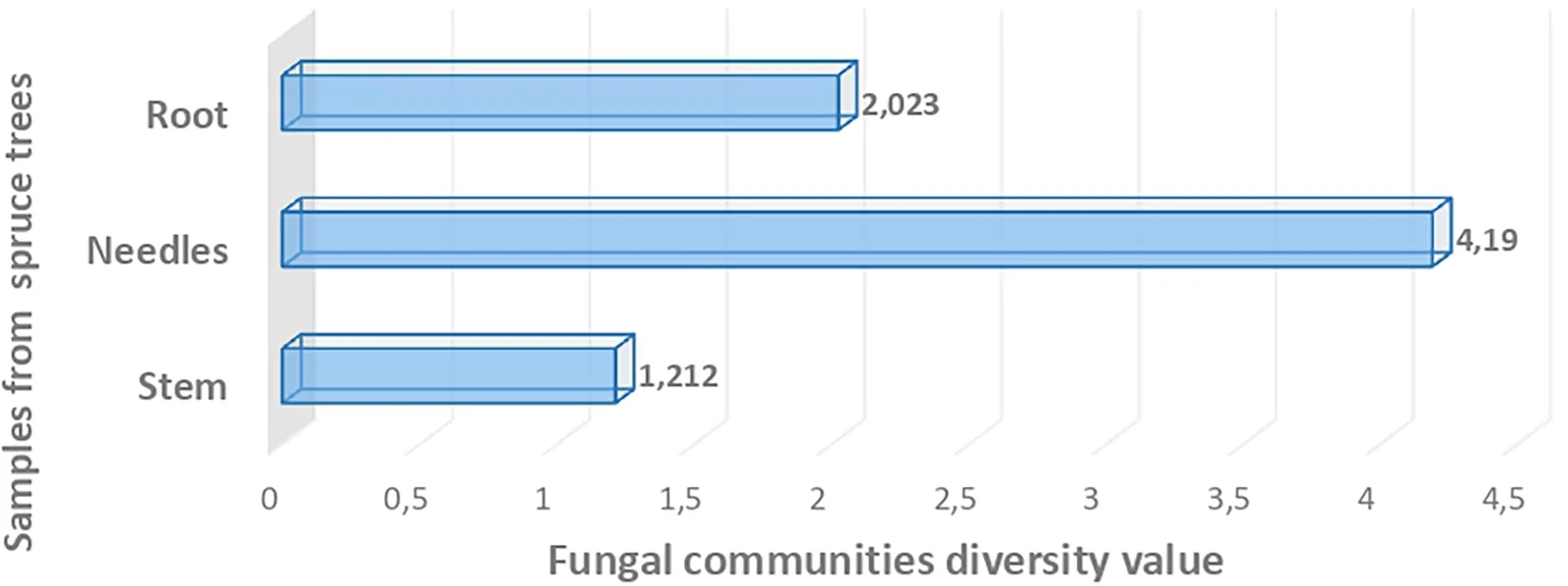
Biodiversity of fungal communities in samples collected from Schrenk's spruce trees, as measured by the Shannon index.

## 4 Discussion

The decline and mortality of Schrenk’s spruce (*Picea schrenkiana*) in the Sary-Chelek Biosphere Reserve appear to result from a complex interaction of biotic and abiotic factors rather than from a single causal agent. Forest decline is increasingly recognized as a multifactorial process in which environmental stress reduces host vigor and predisposes trees to insect infestation and colonization by opportunistic or pathogenic microorganisms (Talley et al., 2002; Lee et al., 2014; Shen et al., 2022; Forkuo and Borz, 2026). The present study supports this framework by demonstrating that declining Schrenk’s spruce harbors tissue-specific fungal assemblages together with evidence of insect infestation. Importantly, the observed fungal diversity should not be interpreted as evidence that all recovered taxa are primary pathogens. Rather, the results suggest a dynamic disease complex in which environmental stress, insect activity, host susceptibility, and microbial colonization may interact during different stages of tree decline.

A pronounced tissue-specific distribution of fungi was observed in declining Schrenk’s spruce. Diseased needles supported the highest fungal diversity, whereas roots and stem bark contained less diverse but compositionally distinct fungal assemblages. Such compartmentalization is ecologically meaningful because different tissues provide contrasting microhabitats in terms of moisture, nutrient availability, chemical composition, structural characteristics, and host defense compounds. Consequently, the fungal assemblages recovered from needles, roots, and bark may reflect different stages and pathways of microbial colonization rather than a uniform fungal community throughout the host. The predominance of *Penicillium* across several host tissues is particularly noteworthy. Members of this genus are generally regarded as saprotrophs or endophytes, although some species can behave as opportunistic pathogens in physiologically weakened or damaged plant tissues (Udagawa and HorieY, 1972; Frisvad and Samson, 2004; Varga et al., 2010). Their repeated recovery from needles, roots, and stem tissues therefore suggests that *Penicillium* may be an important component of the secondary fungal community associated with declining trees. However, the present data do not demonstrate a primary pathogenic role. A more plausible interpretation is that these fungi exploit tissues whose physiological and defensive capacity has already been compromised by drought, insect injury, mechanical damage, or other forms of environmental stress.

*Alternaria alternata* was the second most abundant taxon isolated from symptomatic needles. Species of *Alternaria* are widely recognized as foliar pathogens capable of causing necrotic lesions, chlorosis, and premature senescence under favorable environmental conditions (Rotem, 1998; Nasehi et al., 2012). The occurrence of *A. alternata* in symptomatic spruce needles is therefore of ecological interest because foliar infection may further reduce photosynthetically active tissue in already weakened trees. In a declining conifer, repeated or severe loss of functional needles could potentially contribute to a feedback process in which reduced carbon assimilation further limits host defense and increases susceptibility to additional microbial and insect colonization. The coexistence of *Alternaria* with *Penicillium* also supports the concept of a polymicrobial disease environment rather than a single-agent disease process.

### 4.1 Newly recorded fungal associations and their ecological significance

The isolation of *Curvularia rouhanii* from symptomatic Schrenk’s spruce needles represents an important finding. To our knowledge, this is among the first records of this species associated with declining Schrenk’s spruce in Central Asia. The distinctive lesions observed in association with this fungus suggest that it may occupy a specific ecological niche within the foliar fungal community (Jeon et al., 2015; Manamgoda et al., 2015; Khadka, 2016; Iturrieta-González, et al., 2020). Nevertheless, because information concerning its pathogenicity and host range in conifer ecosystems remains limited, its role should currently be regarded as unresolved. Pathogenicity experiments and re-isolation studies will be required to determine whether *C. rouhanii* is capable of initiating disease or whether it primarily colonizes tissues that have been previously damaged. Similarly, *Didymella glomerata* was recovered from infected needles and shoots, where characteristic dark pycnidia were observed. Members of *Didymella* exhibit diverse ecological strategies, ranging from endophytic and saprotrophic lifestyles to pathogenicity (Deng et al., 2011; Hou et al., 2020; Yuri, 2013). The detection of *D. glomerata* in declining Schrenk’s spruce expands the known fungal diversity associated with this conifer and suggests that this genus deserves greater attention in future forest-health surveys.

Root tissues contained substantially lower fungal diversity than needles but consistently yielded *Penicillium* and *Talaromyces*. The detection of *Talaromyces* is particularly important because this genus has not previously been reported from Schrenk’s spruce in Kyrgyzstan. *Talaromyces* species are widely distributed soil-associated fungi involved in decomposition and nutrient cycling, and some species occur as endophytes or opportunistic pathogens (John, 1979; Wani et al., 2025). Their presence in declining roots may therefore reflect changes in the below-ground microbial community accompanying tree stress. Whether these fungi contribute to root dysfunction or merely exploit already compromised tissues remains an important question for future investigation.

Stem bark supported a distinct fungal assemblage dominated by *Fusarium acuminatum* and *Fusarium avenaceum*. Both species have been associated with stressed plant tissues, and *Fusarium* species can occur in association with vascular discoloration, bark necrosis, and root disorders in forest trees (Lazreg et al., 2013; Vivas and Solla-Hach, 2013). Their predominance in bark suggests that mechanically damaged tissues or tissues affected by insect activity may provide suitable entry points for secondary colonization. This finding is particularly relevant because bark injury may represent an important interface between insect activity and fungal establishment in declining spruce.

### 4.2 Relationship with *Monochamus galloprovincialis* and the pine wilt disease complex

One of the principal objectives of this study was to determine whether the symptoms observed in declining Schrenk’s spruce were associated with the classical pine wilt disease (PWD) complex. PWD is generally characterized by interactions among the pine wood nematode *Bursaphelenchus xylophilus*, *Monochamus* vectors, and associated fungi, including species of *Ophiostoma* and *Ceratocystis* (Donald et al., 2016; Zhao, 2008; Cang-sang, 2011; Vasconcelos and Duarte, 2015). Although adult *Monochamus galloprovincialis* were recovered from declining trees, neither the pine wood nematode nor the characteristic fungal associates of the classical PWD complex were detected. These findings indicate that the widespread mortality of Schrenk’s spruce in the Sary-Chelek Nature Reserve was not associated with pine wilt disease but was primarily linked to severe infestation by longhorn beetles and subsequent colonization by wood-associated microorganisms. This distinction is important for forest-health management. The presence of *M. galloprovincialis* should not, by itself, be interpreted as evidence that classical pine wilt disease is responsible for spruce decline. Instead, the results suggest that insect activity may represent one component of a broader decline syndrome in which damaged or physiologically weakened tissues become more susceptible to secondary fungal colonization. The absence of the classical PWD agents further indicates that the decline observed in Sary-Chelek may involve a different ecological pathway from the well-characterized PWD systems reported elsewhere. Nevertheless, the association between insect occurrence and fungal colonization warrants continued surveillance. Insects capable of boring into woody tissues can create wounds, disrupt vascular tissues, alter local microhabitats, and facilitate the movement of microorganisms between trees. Thus, future forest-health monitoring should consider insects and fungi as interacting components rather than as independent stressors.

### 4.3 Environmental stress as a driver of forest decline

Although the present study focused primarily on biotic components, the observed patterns are consistent with the hypothesis that environmental stress acts as an important predisposing factor. Reduced precipitation, increasing temperatures, and associated drought stress can reduce tree vigor, alter carbon allocation, impair physiological defenses, and increase susceptibility to insects and opportunistic microorganisms. Current climate-related assessments in Kyrgyzstan identify forest ecosystems and biodiversity as vulnerable sectors and emphasize the need to improve forest resilience, monitor pests and diseases, and incorporate climate adaptation into forest management.

This regional context gives particular significance to the findings from Sary-Chelek. Schrenk’s spruce is an ecologically important mountain conifer, and its decline may have consequences extending beyond individual trees. Mountain forests contribute to slope stabilization, soil protection, water regulation, carbon storage, and the maintenance of biodiversity. Consequently, increasing tree mortality may affect multiple ecosystem services simultaneously. The importance of maintaining healthy mountain forests is increasingly recognized in Kyrgyzstan, where current forest and climate initiatives emphasize forest restoration, ecosystem resilience, biodiversity conservation, and improved monitoring of forest pests and diseases.

The present results therefore support a shift from a narrowly pathogen-centered interpretation of spruce decline toward an integrated forest-health framework. Under such a framework, drought and other climatic stresses should be considered predisposing factors, insect activity as a potential facilitating factor, and fungal communities as dynamic components that may include pathogens, opportunists, saprotrophs, endophytes, and antagonists. This conceptual model is more consistent with the complex microbial and ecological patterns observed in the Sary-Chelek Biosphere Reserve.

### 4.4 Implications for forest management and conservation

The findings have several practical implications for the management and conservation of Schrenk’s spruce forests. First, routine forest-health monitoring should combine visual assessment of crown condition and needle symptoms with surveillance for bark- and wood-boring insects and targeted sampling of symptomatic tissues. Because the present study demonstrated clear tissue specificity, sampling needles, roots, and bark separately may provide more informative diagnostic data than analyzing bulk plant material.

Second, molecular identification of fungal isolates can be incorporated into forest-health monitoring to establish a regional baseline of fungi associated with declining trees. Such a baseline would allow future surveys to distinguish persistent components of the spruce microbiome from newly emerging or expanding taxa. In particular, *Curvularia rouhanii, Didymella glomerata* and *Talaromyces*, which were detected in association with Schrenk’s spruce for the first time in the region, should be included in subsequent monitoring efforts.

Third, forest management strategies should place greater emphasis on early detection of declining trees and on identifying environmental conditions that predispose spruce stands to biotic attack. Rather than relying exclusively on the removal of visibly diseased trees, an integrated approach could combine climatic monitoring, assessment of tree vigor, insect surveillance, fungal diagnostics, and targeted sanitation measures. Such an approach would be consistent with the broader direction of climate-resilient forest management in Kyrgyzstan, where forest protection from pests and diseases and improvement of forest ecosystem resilience are identified as important adaptation measures.

The potential role of indigenous *Trichoderma* isolates also deserves attention. If their antagonistic activity observed *in vitro* is confirmed under greenhouse and field conditions, native strains could potentially be developed as environmentally compatible biological agents for reducing the establishment of selected fungal pathogens. Such an approach would be particularly relevant in protected mountain ecosystems where minimizing unnecessary chemical interventions is desirable. However, any biocontrol application should be preceded by rigorous assessment of efficacy, host safety, ecological compatibility, and persistence in the environment.

### 4.5 Limitations and directions for future research

The present study provides an important baseline but does not establish causal relationships between individual fungal taxa and spruce mortality. Isolation frequency, tissue association, and morphological or molecular identification alone cannot distinguish primary pathogens from secondary colonizers, endophytes, or saprotrophs. Similarly, the association between *M. galloprovincialis* and declining trees does not demonstrate that this insect initiates the decline. These limitations highlight the need for experimental studies that move beyond association toward causality.

Future research should therefore integrate seasonal and multi-year field sampling with quantitative assessment of tree health, climatic variables, insect abundance, and fungal community structure. High-throughput sequencing or metabarcoding could provide a more comprehensive characterization of the spruce mycobiome, including unculturable and low-abundance taxa. Pathogenicity assays, re-isolation experiments, and controlled inoculation trials should be used to determine the functional roles of *Alternaria*, *Curvularia*, *Didymella*, *Fusarium*, and other recurrent taxa. Particular attention should be given to *Curvularia rouhanii, Didymella glomerata* and *Talaromyces* because their association with Schrenk’s spruce in Kyrgyzstan appears to be newly documented.

An additional research priority should be to clarify the ecological relationship among climate stress, *M. galloprovincialis*, fungal colonization, and host defense. Long-term monitoring could determine whether periods of drought precede increases in insect infestation and fungal disease, thereby helping to identify early-warning indicators of forest decline. Combining these data with remote sensing and spatial mapping could eventually support risk maps identifying spruce stands most vulnerable to climate-induced decline and biotic disturbance.

### 4.6 Ecological significance and concluding perspective

Overall, the findings support a multifactorial model of Schrenk’s spruce decline in the Sary-Chelek Biosphere Reserve, involving environmental stress, insect infestation, tissue-specific fungal communities, and interactions among potentially pathogenic and antagonistic microorganisms. Rather than identifying a single primary pathogen, this study demonstrates that different fungal taxa are associated with different host tissues and may perform contrasting ecological functions during the progression of tree decline.

The ecological significance of these findings extends beyond the description of fungal diversity. The study provides a baseline for understanding how the microbiome of a keystone mountain conifer responds to tree stress and highlights the need to integrate microbial, insect, and climatic indicators into forest-health assessment. This is particularly relevant in Kyrgyzstan, where strengthening the resilience of forest ecosystems, conserving biodiversity, and improving monitoring of forest pests and diseases are increasingly important components of environmental and climate policy.

In practical terms, the results provide a foundation for developing an integrated monitoring system for Schrenk’s spruce that combines early detection of crown and needle symptoms, insect surveillance, tissue-specific fungal diagnostics, and assessment of climatic stress. The identification of native antagonistic *Trichoderma* isolates additionally opens a potential avenue for environmentally compatible biological protection, although their effectiveness under natural conditions remains to be demonstrated. Previous studies have shown that Trichoderma species parasitizing Fusarium are generally observed after the appearance of this pathogen (Yao et al., 2023).

Future research should consequently move from descriptive characterization toward experimental validation of ecological functions and causal relationships. In particular, determining which microorganisms contribute directly to tissue damage, which merely exploit weakened tissues, and which contribute to natural disease suppression will be essential for developing evidence-based conservation measures. Such knowledge could ultimately support adaptive forest management, early-warning systems, and targeted conservation strategies aimed at maintaining the ecological integrity and climate resilience of Schrenk’s spruce forests in the Tien Shan and other mountain ecosystems of Central Asia.

## Data Availability

The raw data supporting the conclusions of this article will be made available by the authors, without undue reservation.
